# Biomolecular Condensates Act as Distinct Solvation Environments That Reshape Amino Acid p*K*_a_ Values

**DOI:** 10.1021/jacs.6c01118

**Published:** 2026-06-09

**Authors:** Shiv Rekhi, Jeetain Mittal

**Affiliations:** † Artie McFerrin Department of Chemical Engineering, 14736 Texas A&M University, College Station, Texas 77843, United States; ‡ Department of Chemistry, Texas A&M University, College Station, Texas 77843, United States; § Interdisciplinary Graduate Program in Genetics and Genomics, Texas A&M University, College Station, Texas 77843, United States

## Abstract

Biomolecular condensates create distinct solvation environments in which the ionization equilibria of amino acid side chains may differ markedly from those in a bulk aqueous solution. Here, we use all-atom continuous constant pH molecular dynamics simulations to investigate the changes to the p*K*_a_ values of titratable residues between the coexisting phases of biomolecular condensates. We found that protonated states are favored in the condensate, resulting in the stabilization of charged forms of cationic residues and neutral forms of anionic residues. The effect is consistent across condensates formed by five peptide sequences, suggesting that the preference for protonated states is a general feature of the condensate microenvironment. These results highlight that differences in solvation environments between coexisting phases are key determinants of charge regulation in phase-separating proteins.

## Graphical abstract

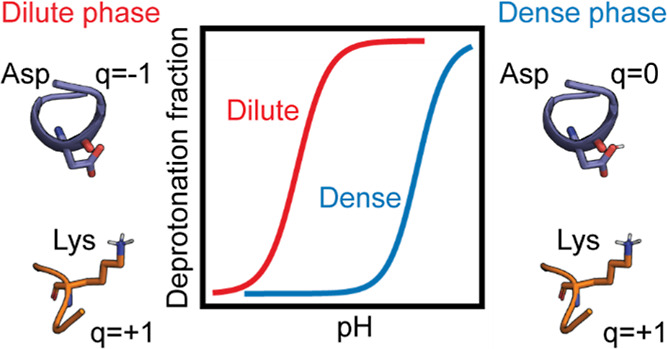

## Introduction

Biomolecular condensates represent a distinct physicochemical phase of matter in which proteins experience environments that differ fundamentally from bulk aqueous solution.^1^ To maintain electroneutrality, charged phase-separating proteins sequester counterions within the dense phase, shaping a unique electrostatic and solvation environment.^2–4^ While phase separation is often discussed in terms of concentration-driven organization, the dense phase also imposes altered solvation,^5,6^ dielectric response,^7,8^ and electrostatic screening^9^ that can directly reshape chemical equilibria. A central but largely unexplored consequence of this altered environment is its impact on charge regulation,^10^ which depends sensitively on the solution pH and the p*K*_a_ values of titratable amino acid side chains. Although recent studies have reported shifts in the pH of the dense phase,^11,12^ these changes alone are insufficient to account for the substantial modulation of protein charge expected within condensates,^11^ suggesting that the intrinsic p*K*_a_ values of amino acid side chains may themselves be altered^13,14^ within the condensate microenvironment.

Previous work on charge regulation in disordered proteins^10,15^ and polyelectrolytes^16–19^ has involved the use of implicit solvent or coarse-grained models, which successfully capture pH-dependent effects originating from the electrostatic interactions between monomers. However, solvent reorganization, which requires an explicit representation of solvent molecules, can dominate the charging free energy of a titratable residue.^20,21^ Therefore, we use all-atom continuous constant pH molecular dynamics (AA CpHMD) simulations^22^ with explicit solvent^23,24^ to calculate the p*K*_a_ values of four titratable amino acid side chains (Asp, Glu, His, and Lys) within the dense phase. In AA CpHMD, λ-dynamics^25^ is used to sample protonation states along a pH-dependent free energy surface. The parameters of this surface are calibrated to match the experimental p*K*_a_ values of the amino acid embedded within a model pentapeptide (ACE-AAXAA-NHE) in solution. By calculating the p*K*_a_ values of the same pentapeptide placed within a model condensate, we provide an estimate of the p*K*_a_ shifts due to the condensate microenvironment relative to the dilute (aqueous) phase.

Our calculations reveal that the p*K*_a_ values of both anionic and cationic amino acids are shifted up in the condensate relative to the dilute phase, suggesting that protonated states are stabilized in the condensate microenvironment. This finding is consistent across five different model condensate systems with varying sequence features, such as net charge, aliphatic amino acid content, and polar amino acid content. This suggests that the favorability of protonated states within condensates shows only weak sequence dependence and primarily originates from the condensate solvation environment. Because of the shifted p*K*_a_ values, the net charge of a given protein sequence within a condensate can be significantly different from expectations based on dilute-phase model p*K*_a_ values. These findings uncover a distinct feature of the solvation environment of biomolecular condensates, with broad implications for understanding the sequence-encoded electrochemical environment and function of these assemblies.

## Methods

### Preparation and Equilibration of Condensate Systems

To generate condensate systems for the CpHMD simulations, first, a single copy of the scaffold sequence (SYGQ, APGVG, GRGDSPYS, GRGNSPYS, or GQGDSPYS) in pdb format was prepared using the tleap module in AmberTools.^26^ Following this, PACKMOL^27^ was used to place multiple copies of the scaffold protein within a 5 nm side cubic box such that the protein concentration within the simulation cell was within the range of the experimentally determined concentrations of FUS LC^28^ and DDX4 NTD^29^ condensates. The system was then solvated, and ions were added to achieve a salt concentration of 150 mM. The systems were modeled using AMBERFF14SB^30^ (with NBFix corrections^31^) for proteins and the TIP3P water model^32^ was used. Hydrogen masses were repartitioned^33^ by a factor of 1.5 to enable a time step of 4 fs. The system was then energy minimized and equilibrated in a series of steps which involved heating the system to the target temperature with position restraints applied on the protein chains, slowly releasing the restraints, and equilibrating the pressure (runtimes and run parameters for each step provided in Supporting Information). The final NPT step was then extended to 1 μs to adequately equilibrate the chains within the condensate. Coordinates were written to the trajectory file every 100 ps for analysis.

The density profiles from the above simulation with the AMBER14SB + TIP3P force fields showed that proteins clustered within the simulation box (Figure S1). To prevent the aggregation of the protein chains into a phase of higher concentration within the simulation box, we scaled the protein-water Lennard-Jones (LJ) interactions^34^ by a factor of 1.1. We used the same initial solvated configuration of peptide chains and used the Parmed module in AmberTools^26^ to apply the scaling of LJ interactions. The system was equilibrated following the same steps as detailed above and the density of protein within the simulation box was calculated from a 1 μs simulation. The density profile showed an improvement compared to the unscaled AMBER14SB + TIP3P water model (Figure S1). Consistent with previous simulation studies^5^ using model condensate systems and atomistic simulations of IDR condensates, we observed fluctuations in the protein concentration along the *z* dimension of the simulation box of the order of ∼100 mg/mL. This suggests that there are regions of higher or lower protein concentration within the simulation box. Given the improvement in the density profiles of the system, the AMBER14SB + TIP3P water model with protein-water LJ interactions scaled by 1.1 times was used for all the following simulations in this work.

### All-Atom Continuous Constant pH Molecular Dynamics Simulations

All-atom continuous constant pH molecular dynamics (AA CpHMD) simulations sample the time evolution of protonation states of a titratable residue using λ-dynamics.^23,35^ In AA CpHMD, the titration coordinates (λ for single site titration and λ and *x* in double site titration) are propagated using an extended Hamiltonian. The λ and *x* parameters are alchemical coordinates that interpolate between the protonated (λ = 0) and deprotonated states (λ = 1) and, if applicable, different tautomeric states (*x* = 0, *x* = 1) of the titratable residue. Details of the method have been described elsewhere.^36,37^

The pH dependence of the titration coordinates is included through biasing potentials that ensure appropriate pH-dependent sampling with respect to a reference solvent condition. Here, the parameters for these biasing potentials are calibrated such that the p*K*_a_ of the titratable residue embedded within a model pentapeptide of sequence AAXAA, where X is the titratable residue, in aqueous solution matches the experimentally determined p*K*_a_ value. The AAXAA peptide was selected as the model peptide system in this work for two reasons. First, the availability of experimental data for the four titratable residues in the dilute phase allows for precise tuning of the additional biasing potentials to recover the experimental values. Second, in our work, our primary interest is to quantify the effect of the microenvironment within the condensate on the p*K*_a_ of the titratable amino acids. Therefore, we use the AAXAA peptide, as it minimizes sequence context and conformation-dependent effects on the p*K*_a_ estimates. Parameter files for the commonly used force fields and water models, such as the AMBER14SB force field and TIP3P water model used in this work, are available on the JanaShenLab GitLab page.^23,38^

As the available parameter sets are generated based on the default AMBER14SB + TIP3P water model combination, we first evaluated the effect of protein-water LJ interaction scaling on the dilute-phase p*K*_a_ values. The systems were prepared (see the following section on the preparation of systems for CpHMD simulations), equilibrated (details provided in the Supporting Information), and AA CpHMD simulations were performed to estimate p*K*_a_ values (see the following sections on the analysis of CpHMD simulations).

We find that the p*K*_a_ values between the scaled and unscaled force field variants were within the statistical uncertainty for all of the residues (Figure S2, Table S1). Thus, we did not make any changes to the original CpHMD parameter file downloaded from the JanaShenLab GitLab page. The p*K*_a_ values of the titratable residues from these simulations with the scaled protein-water interactions were used as the reference dilute-phase values to compute the p*K*_a_ shifts due to the condensate microenvironment.

### Preparation of Systems for CpHMD Simulations

To prepare systems for CpHMD simulations in Amber, two additional force field files are required. First, a .lib file which contains residue definitions for protonated forms of ASP (named AS2) and GLU (named GL2). Second, an frcmod file which contains parameters for bonds and dihedrals for titratable residues. These files were downloaded from the JanaShenLab GitLab page.

For the dilute-phase simulations, tleap was used to generate a structure of the model pentapeptide (AAXAA) within a cubic water box of side 5 nm (Figure 1). Separate systems were prepared for each titratable residue considered (Asp, Glu, His, and Lys). NBFix corrections were applied in this step, and counterions were added on the basis of the charge of the residue at neutral pH. Therefore, one Na^+^ ion was added in the Asp and Glu systems, and 1 Cl^–^ ion was added in the Lys system. The parmed module of AmberTools was then used to repartition the hydrogen masses by a factor of 1.5 and scale the protein-water interactions by a factor of 1.1. Following this, the system was equilibrated, and then the production run was conducted with a time step of 4 fs. For the production runs, 12.5 ns of asynchronous pH replica exchange simulations^39^ were conducted for each titratable residue.

**Figure 1:**
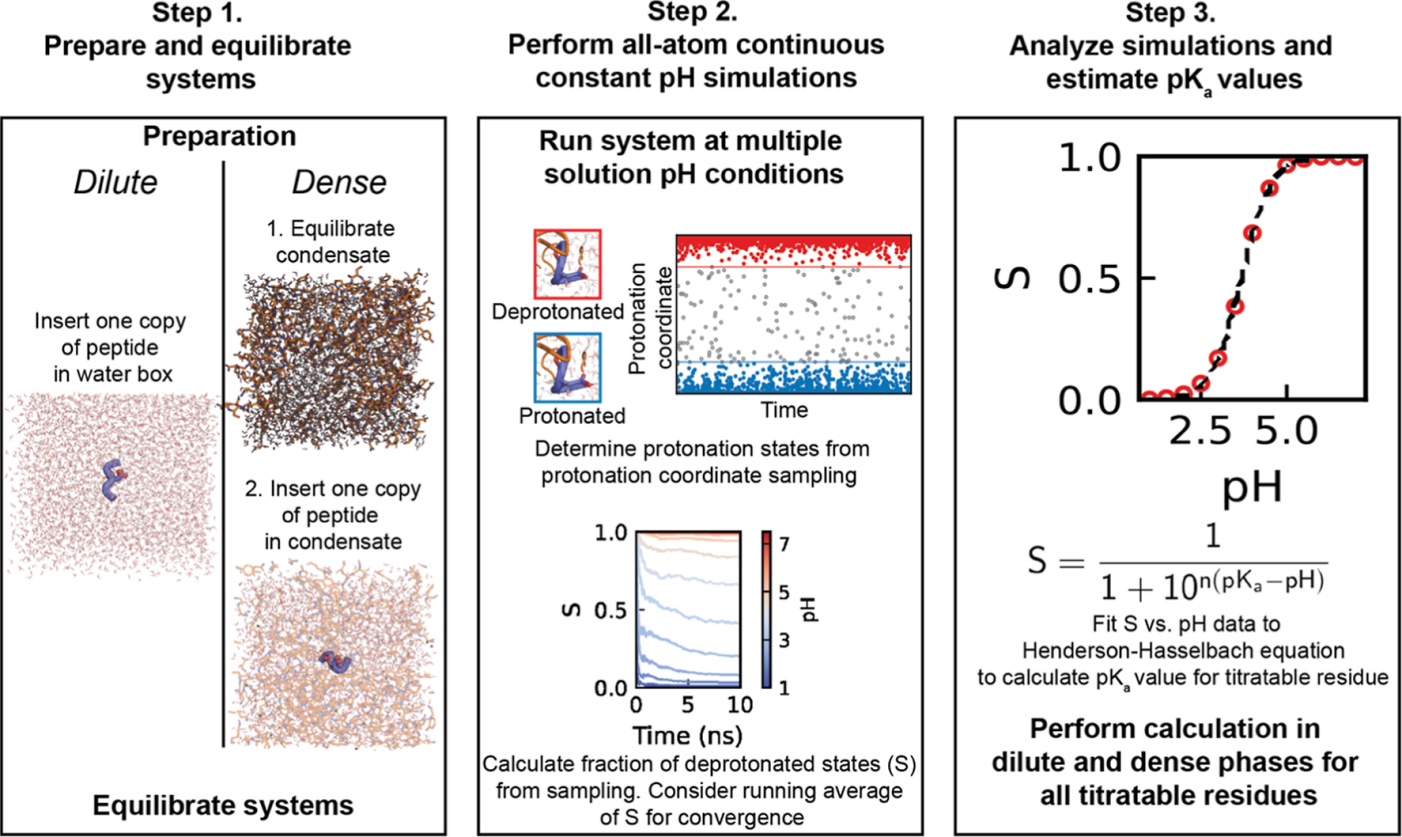
Workflow for calculating p*K*_a_ values in AA CpHMD simulations. Systems are prepared and equilibrated: the model pentapeptide is inserted into a water box for the dilute phase and into a pre-equilibrated condensate for the dense phase. Simulations are performed across multiple pH values, and the protonation coordinate is used to obtain the fraction of deprotonated states (S) and assess convergence. Converged S values are used to construct titration curves, which are fit to the Henderson–Hasselbalch equation to extract p*K*_a_ and the Hill coefficient (n). The same protocol is applied to all residues in both phases. Data in steps 2 and 3 correspond to Asp (AADAA) in the dilute phase.

For the dense-phase simulations, the final frame from the 1 μs long NPT simulations of the condensate systems was extracted. One copy of the model pentapeptide containing the titratable residue was inserted into this pre-equilibrated snapshot using the GROMACS^40^ gmx_insert-molecules command, allowing for the replacement of overlapping water molecules, and a pdb file was written (Figure 1). The residue names in the pdb file were edited to match the definitions in the .lib file and then the file was passed to the tleap module to write out the structure and topology files for simulations in Amber. Counterions were added following the same protocol as for the dilute phase. Parmed was used to scale the protein-water interactions by 1.1 and repartition the hydrogen masses by a factor of 1.5 to allow for a time step of 4 fs. The systems were then equilibrated and production runs of 20 ns of asynchronous pH replica exchange simulations were conducted.

### Analysis of CpHMD Simulations

To calculate the p*K*_a_ values of a titratable residue, the system is run at multiple solution pH values and the fraction of deprotonated states at each pH value, denoted by S is calculated (Figure 1). These S values are then fit to the Henderson–Hasselbalch (HH) equation (Figure 1), shown below, to calculate the p*K*_a_ values and the Hill coefficient (n) of the titratable group.

1 $$S=\frac{1}{1+10^{n\left(pK_{a}-pH\right)}}$$

Values of *n* < 1 and *n* > 1 indicate anticooperativity and cooperativity, respectively.^35^ S values are calculated considering λ < 0.2 and λ > 0.8 as protonated and deprotonated states and *x* < 0.2 and *x* > 0.8 for tautomeric states, respectively.^23,41^ Intermediate alchemical states in the range of 0.2–0.8 are ignored from the calculation of S.

To improve the sampling of the titration coordinates, we used the asynchronous pH replica exchange sampling scheme.^39^ Details of the pH ranges used for each of the titratable groups in the dilute phase and dense phase simulations are provided in the Supporting Information. We consider the running average of S during the simulation runtime to judge convergence of the simulations (Figure 1). For our analysis, we ignore the initial equilibration of S and then divide the remaining trajectory into 3 equally spaced blocks. We calculate the p*K*_a_ value of the titratable residue in each block and then report the mean as the final p*K*_a_ of the titratable residue and the standard error of the mean across the 3 blocks as the uncertainties. In the dilute phase, the simulations were run for 12.5 ns with the first 3.125 ns ignored from the analysis as equilibration of the titration coordinates (Figure 1).

Achieving reliable sampling of the titration coordinates in the dense phase is more challenging due to the heterogeneous environment consisting of both protein and water. We find that within 20 ns of replica exchange simulations, replicas exchange freely (Figure S3) and converged estimates of S are obtained within the dense phase for all titratable residues. The running average of the S values begins to plateau after an equilibration phase of ∼5 ns (Figure 2). Therefore, we ignore the first 5 ns of sampling and use the remainder of the trajectory for block averaging to estimate uncertainties in the p*K*_a_ values.

**Figure 2:**
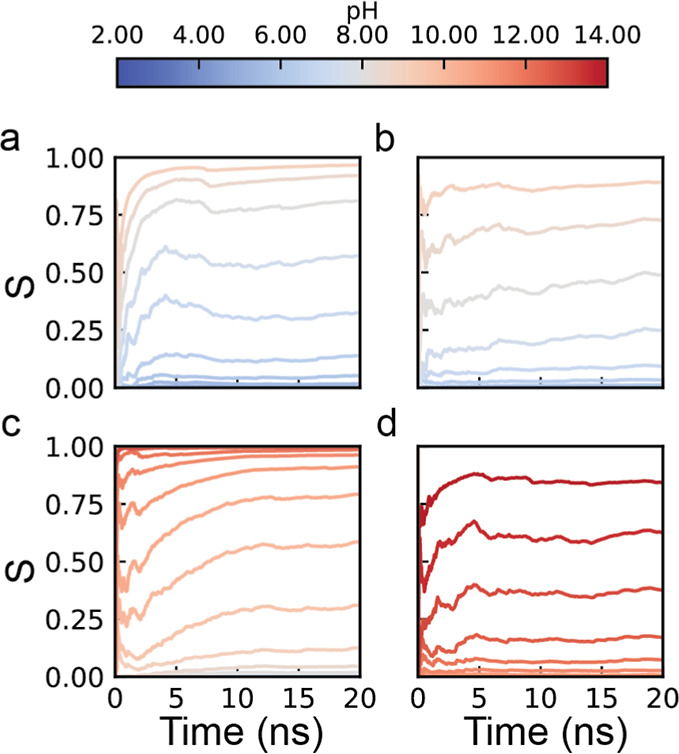
20 ns of asynchronous pH replica exchange simulations lead to converged S values in the dense phase. Running average of deprotonated fraction (S) with time at different solution pH values for the (a) Asp, (b) Glu, (c) His, and (d) Lys systems within the SYGQ condensate.

## Estimates of Dielectric-Dependent Desolvation and Background Contributions to p*K*_a_ Shifts of Titratable Residues

We calculate the p*K*_a_ shifts (Δ*pK*_a_) of the titratable residues between the dilute and the dense phase and then convert these estimates into charging free energy differences^13^ between the media by,

2 $$\Delta G_{charging}=\left(z\right)2.303RT\left(\Delta pK_{a}\right)$$

where Δ*pK*_*a*_ is given by p*K*_a_^dense^–p*K*_*a*_^dilute^ and *z* is +1 for anionic residues and −1 for cationic residues to reflect the trend of anionic residues being charged above their p*K*_a_ and cationic residues being uncharged above their p*K*_a_.

We decompose this charging free energy into two terms to reflect the Born-like dielectric- and size-dependent desolvation term and contributions beyond the bulk dielectric response of the medium, such as local solvent rearrangement, hydrogen bonding, and protein-mediated interactions, collectively referred to as “background” interactions.^20,21,42^

3 $$\Delta G_{charging}=\Delta G_{Born}+\Delta G_{background}$$

The Born desolvation term is given by,

4 $$\Delta G_{Born}=\frac{-z^{2}e^{2}}{8\pi \epsilon_{0}r}\left[\frac{1}{\epsilon_{dilute}}-\frac{1}{\epsilon_{dense}}\right]$$

where *z* is the charge, *e* is the elementary charge, ϵ_0_ is the vacuum permittivity, *r* is the radius of the ion, and ϵ_dense_ and ϵ_dilute_ are the dielectric constants of the condensate and water, respectively.

For the radii of the residues, we use half the van der Waals diameters used in single-bead-per-amino-acid resolution coarse-grained models.^43^ As we consider the dilute phase to be pure solvent, we use the dielectric constant of a pure TIP3P water box as ϵ_dilute_ = 98 and the SYGQ condensate system as ϵ_dense_ = 66.2. We substitute these values along with the radii of the residues in eq 4 to obtain the Born-like term for each titratable residue and then estimate the background contributions as Δ*G*_charging_–Δ*G*_Born_.

### Calculation of Net-Charge-Per-Residue Profiles as a Function of pH

We computed the net-charge-per-residue for the four IDP sequences in the dilute and dense phase as a function of pH using the HH equation, substituting the p*K*_a_ values of the titratable residues measured in this work. The experimentally determined p*K*_a_ values for Asp, Glu, His, and Lys, shown in Table S1, were used as the model p*K*_a_ values in the dilute phase, while the p*K*_a_ values of the residues in the SYGQ condensate shown in Table 1 were used as the dense phase p*K*_a_ values. We considered Arg to always be charged within the pH range investigated. We did not consider the possible changes in the charge state arising from other titratable groups such as the backbone, tyrosine, and cysteine in our calculations. The range of pH values designated as biologically relevant spans from 4.5 to 8, representative of the pH of the lysosome (4.5–5.0) and the mitochondrial matrix and some membraneless compartments (∼8).

**Table 1:** p*K*_a_ Values in the Dilute and Dense Phase of the SYGQ Condensate and p*K_a_* Shifts (Δ*pK*_*a*_) for Asp, Glu, His, and Lys Residues

residue	p*K_**a**_^**dilute**^*	p*K_a_^dense^*	Δ*pK*_*a*_
**Asp**	3.73 ± 0.02	7.36 ± 0.10	3.63 ± 0.10
**Glu**	4.35 ± 0.06	7.98 ± 0.07	3.63 ± 0.09
**His**	6.65 ± 0.10	9.76 ± 0.03	3.11 ± 0.10
**Lys**	9.96 ± 0.06	13.25 ± 0.06	3.30 ± 0.09

## Results and Discussion

### Neutral Forms of Anionic Amino Acid Side Chains Are Favored within the Condensate

Following our prior work^5^ demonstrating the validity of minimal peptide systems as model condensate systems, we consider the dense phase formed by the minimal Ser-Tyr-Gly-Gln (SYGQ) peptide unit as our model system. The protein and water densities within the condensate were set close to the NMR estimates of the FUS LC condensate,^28^ and the system was equilibrated. Following the equilibration, titration curves of the residues were obtained by using AA CpHMD simulations.

In the condensate, the titration curves for Asp and Glu are shifted toward more basic conditions, yielding p*K*_a_ values of 7.36 ± 0.10 and 7.98 ± 0.07, respectively (Figure 3, Table 1). The upshifts of 3.6 units for both Asp and Glu in the condensed phase (Table 1) indicate that the protonated neutral form of these amino acids is favored within the dense phase. Similar magnitudes of p*K*_a_ shifts for Asp have been reported in CpHMD simulations of transmembrane helices.^14^ Substituting the p*K*_a_ shifts of 3.63 units for Asp and Glu in eq 2, we find that the charging free energies (Δ*G*_charging_) for both Asp and Glu are unfavorable with values of 4.98 ± 0.14 kcal/mol and 4.98 ± 0.12 kcal/mol, respectively. The positive free energy of charging within the condensed phase compared to the dilute phase follows from the expectation of a reduced dielectric constant of 66.2 within the dense phase^7,8^ of the SYGQ condensate. We calculate the contribution originating from the difference in dielectric constants (Δ*G*_Born_) using the Born solvation model. Substituting the values for the dielectric constants of the dilute phase and dense phase, we find that Δ*G*_Born_ is 0.29 and 0.27 kcal/mol for Asp and Glu, respectively. The positive signs of both Δ*G*_charging_ and Δ*G*_Born_ indicate that the reduction in dielectric constant within the condensed phase qualitatively describes the p*K*_a_ shifts within the condensate for the anionic residues. However, Δ*G*_*Born*_ is an order of magnitude lower than Δ*G*_charging_, highlighting that the dielectric constant of the dense phase alone cannot describe the observed differences in Δ*G*_charging_ or Δ*pK*_*a*_.

**Figure 3:**
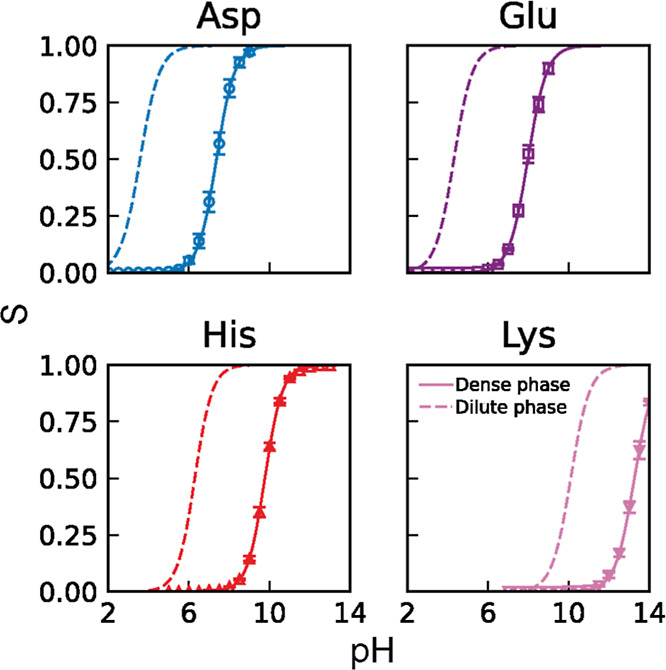
Anionic and cationic residues show elevated p*K*_a_ values in the dense phase compared to the dilute phase. Titration (S vs pH) curves for Asp, Glu, His, and Lys residues in the dilute phase (dashed lines) and dense phase (symbols and solid lines). For the dilute phase, only the HH equation fits are shown. For the dense phase, symbols represent the S values estimated from simulation and solid lines show the HH fit to the simulation data. Uncertainties were estimated as the SEM over 3 equally spaced 5 ns blocks.

To elucidate the remaining contributions to Δ*pK*_*a*_, we quantify the background interactions (Δ*G*_background_) as Δ*G*_charging_–Δ*G*_Born_. For both Asp and Glu, Δ*G*_background_ is positive and significantly larger than Δ*G*_Born_ with values of 4.69 and 4.71 kcal/mol, respectively. Therefore, for the anionic residues, both the dielectric-dependent Born term and the background interactions favor the uncharged state within the condensate. However, the dominant contribution to the overall unfavorable charging free energy is the unfavorable background interactions indicating that condensates cannot be treated as simple low-dielectric constant continua.

### Charged Forms of Cationic Amino Acid Side Chains Are Favored within the Condensate

Like Asp and Glu, the titration curves for the cationic residues are shifted to higher pH in the condensate and yield p*K*_a_ values of 9.76 ± 0.03 and 13.25 ± 0.06 for His and Lys, respectively (Figure 3, Table 1). The positive Δ*pK*_a_ values of 3.30 for Lys and 3.11 for His (Table 1) reflect a preference for the positively charged protonated forms within the condensate. Δ*G*_charging_ is negative for the cationic amino acids with Lys (−4.53 ± 0.14 kcal/mol) being marginally more favorable than His (−4.27 ± 0.12 kcal/mol). The difference in the sign of Δ*G*_charging_ for anionic and cationic amino acids suggests that positively charged amino acids are favored within the condensate over negatively charged amino acids. Computational^5^ and experimental^44^ estimates of the transfer free energies of charged amino acids and ions^45^ from the dilute to the dense phase report favorable transfer of positively charged species and unfavorable transfer of negatively charged species. The stabilization of protonated states observed here provides mechanistic insight on how charge regulation and transfer free energies jointly dictate the solvation of titratable molecules in condensates. Shifting the protonation equilibria of anions to favor neutral forms allows for their incorporation within the condensate microenvironment despite the unfavorable transfer of their charged forms. Conversely, charged forms of cations are stabilized, reflecting their thermodynamically favored incorporation into the dense phase.

The Δ*G*_Born_ terms are unfavorable for both amino acids (0.27 kcal/mol for His, 0.26 kcal/mol for Lys) consistent with the expectation of unfavorable charging in a medium of reduced dielectric constant compared to water. The opposite signs of Δ*G*_charging_ and Δ*G*_Born_ in the case of cationic amino acids highlights that, unlike in the case of anionic residues, the reduced dielectric constant within the condensate does not explain the charging free energies even qualitatively. The positive Δ*G*_Born_ and negative Δ*G*_charging_ for the cationic residues means that Δ*G*_background_ for both His and Lys is highly favorable. The Δ*G*_background_ values of −4.79 kcal/mol for Lys and −4.54 kcal/mol for His reflect these highly favorable background interactions that overcome the dielectric-dependent desolvation penalty and lead to favorable Δ*G*_charging_.

The signs of Δ*G*_background_ for the cationic and anionic amino acids indicates that interactions with the environment favor the charged forms of cationic residues and the neutral forms of anionic residues at the concentrations of protein and water used in this work. However, changes to the solution conditions can alter the composition of the dense phase.^46^ Based on our analysis, we expect that decreasing protein concentrations will increase the dielectric constant of the condensate, leading to less unfavorable Δ*G*_Born_ and reduce the contribution of Δ*G*_background_ due to fewer protein-mediated interactions leading to reduced p*K*_a_ shifts for both cationic and anionic residues in the condensate with respect to the dilute phase. On the other hand, increasing the protein concentration will further reduce the dielectric constant of the condensate, making Δ*G*_Born_ more unfavorable and increase the contribution of Δ*G*_background_. Consequently, we expect the p*K*_a_ shifts for the anionic residues to increase further within the condensed phase. For the cationic residues, we expect an increase in the p*K*_a_ shifts with increasing concentration of protein until the limit where the unfavorable Δ*G*_Born_ term overcomes the favorable Δ*G*_background_ term.

### p*K*_a_ Shifts within the Condensate Are Primarily Dictated by the Solvation Environment

The observation that the dominant contribution to Δ*G*_charging_ for both cationic and anionic residues is Δ*G*_background_, which is favorable for cationic residues and unfavorable for anionic residues, suggests that the sequence of the condensate-forming protein may alter the p*K*_a_ values of the titratable residues. To investigate this possibility, we compute the p*K*_a_ values of the residues in four additional condensate systems. We note that the changes to the protein sequence may alter the dielectric constant of the condensate and therefore the Δ*G*_Born_ term, but given the order of magnitude difference between Δ*G*_background_ and Δ*G*_Born_, we expect the primary effect on the p*K*_a_ values to be through modulation of the protein-mediated background interactions encoded by the sequence. We therefore quantify the p*K*_a_ shifts of the titratable residues in condensates formed by four additional peptide sequences, (i) a variant^47^ of the elastin-like polypeptide (APGVG), (ii) the resilin-like polypeptide^48^ (RLP; GRGDSPYS), (iii) a positively charged RLP variant^49^ (GRGNSPYS), and (iv) a negatively charged RLP variant^49^ (GQGDSPYS).

We find that between all systems the qualitative trend of favorable protonation within the condensed phase is preserved (Figure 4, Table 2, Figure S4). Moreover, the magnitude of Δ*pK*_*a*_ for all residues are relatively consistent across all systems investigated in this work indicating that the observed p*K*_a_ shifts are weakly sequence-dependent (Figure 4, Table 2).

**Figure 4:**
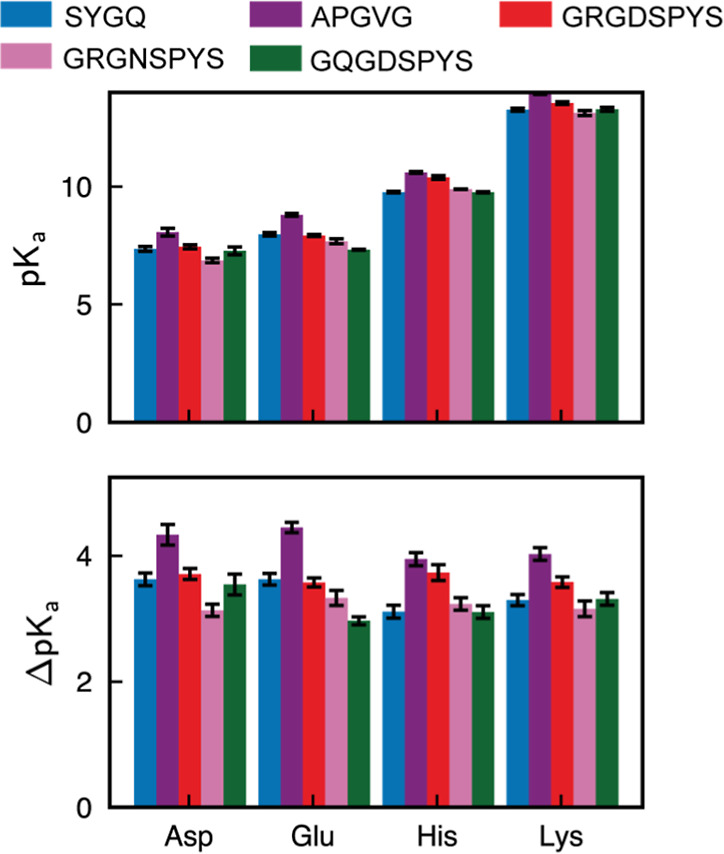
Significant stabilization of protonated states is observed across 5 condensate systems. p*K*_a_ values (upper panel) and p*K*_a_ shifts with respect to the dilute phase (lower panel) for Asp, Glu, His, and Lys in condensates formed by the SYGQ, APGVG, GRGDSPYS, GRGNSPYS, and GQGDSPYS peptide sequences. p*K*_a_ and Δ*pK*_a_ values for the SYGQ condensate are identical to those in Table 1. Uncertainties for all residues and condensate systems are estimated as the SEM over 3 equally spaced 5 ns blocks. The consistency of the p*K*_a_ shifts across chemically distinct condensates highlights a general solvation-driven effect of the dense phase rather than sequence-specific interactions.

**Table 2:** p*K*_*a*_ Values and p*K*_*a*_ Shifts with Respect to the Dilute Phase (Δ*pK*_*a*_) for Asp, Glu, His, and Lys Residues in the APGVG, GRGDSPYS, GRGNSPYS, and GQGDSPYS Condensates

residue	p*K_**a**_^**APGVG**^*	*ΔpK_**a**_^**APGVG**^*	p*K*_***a***_^**GRGDSPYS**^	Δ*pK*_***a***_^***GRGDSPYS***^	p*K*_***a***_^***GRGNSPYS***^	Δ*pK*_***a***_^***GRGNSPYS***^	p*K*_***a***_^***GQGDSPYS***^	*ΔpK_**a**_^**GQGDSPYS**^*
**Asp**	8.07 ± 0.16	4.33 ± 0.16	7.44 ± 0.08	3.71 ± 0.09	6.87 ± 0.09	3.13 ± 0.10	7.28 ± 0.16	3.54 ± 0.16
**Glu**	8.80 ± 0.06	4.45 ± 0.08	7.93 ± 0.04	3.57 ± 0.07	7.68 ± 0.10	3.33 ± 0.12	7.32 ± 0.02	2.97 ± 0.06
**His**	10.60 ± 0.04	3.95 ± 0.10	10.38 ± 0.08	3.73 ± 0.13	9.89 ± 0.01	3.24 ± 0.10	9.76 ± 0.02	3.11 ± 0.10
**Lys**	13.99 ± 0.08	4.03 ± 0.10	13.54 ± 0.05	3.58 ± 0.08	13.11 ± 0.11	3.16 ± 0.12	13.27 ± 0.08	3.31 ± 0.10

Compared to SYGQ, in APGVG we see higher upshifts for both the anionic and cationic amino acids (Figure 4, Table 2). This suggests that the absence of polar side chains in the peptide sequence leads to further destabilization of the ionized negative form for anions and stabilization of the positive form for cations. Between SYGQ and GRGDSPYS, we observe smaller differences in the p*K*_a_ shifts (Figure 4, Table 2), except for His where in GRGDSPYS, the charged form is marginally stabilized, implying that the addition of charged residues has a minimal effect. Perhaps most surprisingly, the preference for protonated states within the condensate compared to the dilute phase persists even in GRGNSPYS and GQGDSPYS (Figure 4, Table 2). Relative to SYGQ, in GRGNSPYS we see that the associative charging behavior seen in complexation of polyelectrolytes,^17–19^ where p*K*_a_ values shift to minimize charge repulsion and maximize charge interactions, is qualitatively reflected in the mean values of all amino acids. However, the effect is less pronounced than expected (Figure 4, Table 2). In the case of GQGDSPYS, we see that the expectation of upshifted p*K*_a_ values with respect to SYGQ is not reflected even in the mean values (Figure 4, Table 2).

To rationalize the sequence-dependent changes in the p*K*_a_ values of the amino acids, we evaluated whether sequence-based metrics, such as average hydropathy estimated from the Urry hydrophobicity scale,^43,50^ net charge (NC), fraction of charged residues (FCR), and fraction of aromatic residues (FAR) of the five peptide sequences can explain the magnitudes of the observed p*K*_a_ shifts. We observe minimal correlation among average hydropathy, NC, and FCR and the p*K*_a_ shifts for the different amino acids (Figure S5). However, we observe a moderate correlation, particularly in the case of the cationic residues, with FAR (Figure S5). This correlation primarily originates from the APGVG (FAR = 0) and SYGQ (FAR = 0.25) peptide systems at the extremes. Despite this apparent correlation, we see that the GRGDSPYS, GRGNSPYS, and GQGDSPYS peptide systems all have the same FAR values but show different p*K*_a_ shifts for all residues, highlighting that FAR is not adequate to explain the observed preference for protonated states. The weak sequence dependence of the p*K*_a_ shifts and their minimal correlation with sequence features of the condensate-forming protein indicate that the preference for protonated states within the condensate is primarily governed by the solvation characteristics of the dense phase rather than sequence-specific effects.

This minimal sequence dependence of the condensate microenvironment compared to the dilute phase has previously been reported in the investigation of the partitioning of a library of small molecules into four different condensate systems.^51^ Despite some sequence-specific effects, partition coefficients across the library of molecules were correlated among systems. Additionally, previous work^5^ on the transfer free energies of amino acids from the dilute phase to the condensed phase reveals that across a variety of condensate systems, the transfer of positively charged species is favorable, while for negatively charged species it is unfavorable. Together with these studies, our results report another feature of the condensate microenvironment, wherein the protonated states of both anionic and cationic amino acids are favored.

### p*K*_a_ Shifts within the Condensate Significantly Alter the Charge Profiles of IDRs as a Function of Solution pH

The range of p*K*_a_ values observed in the different systems for Asp (6.87–8.07), Glu (7.32–8.80), and His (9.76–10.60) indicates that small pH shifts in the dense phase can significantly alter the net charge on proteins. Therefore, we compute the net-charge-per-residue (NCPR) profiles of four charge-rich IDRs (hnRNPA1-LCD, DDX4-NTD, LAF1-RGG, and RLP with R-to-K mutations) as a function of pH (Figure 5). Within biologically relevant range of pH values of 4.5–8, we see that all the proteins have a positive NCPR (Figure 5). Accounting for the p*K*_a_ shifts in the condensates also leads to a higher magnitude of NCPR across the pH range than expected from the model p*K*_a_ values (Figure 5).

**Figure 5:**
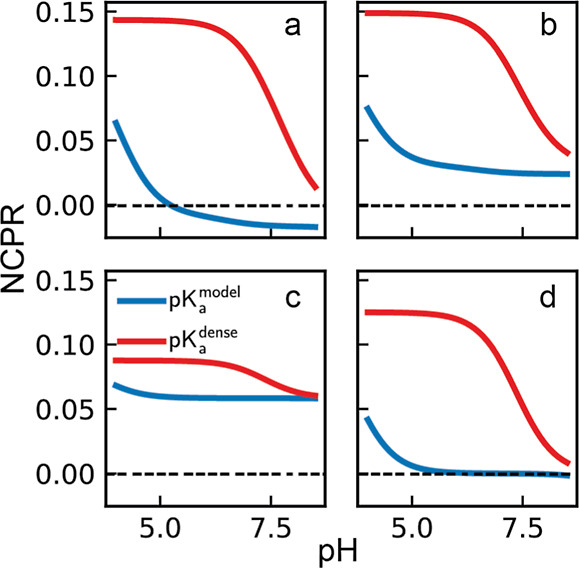
p*K*_a_ shifts in the dense phase lead to positively charged proteins across a wider range of pH values. Net charge per residue (NCPR) as a function of solution pH for (a) DDX4-NTD, (b) LAF1-RGG, (c) A1-LCD, and (d) an RLP variant with all R mutated to K. Blue lines show profiles when using model compound (aqueous) p*K*_a_ values shown in Table S1. Red lines show profiles calculated using the dense phase p*K*_a_ values shown in Table 1. The charge on Arg is always taken as +1 within the range of pH values considered. Cys, Tyr, and the backbone titratable groups are all considered neutral. Sequences and counts of anionic and cationic residues are provided in the Supporting Information.

Recent work from Ausserwöger et al.^12^ reports that the interior pH of biomolecular condensates shifts toward, but does not coincide with, the isoelectric point of the condensate-forming protein to minimize charge repulsion in a mechanism termed charge neutralization. In their work, the authors use aqueous p*K*_a_ values to determine the isoelectric point of the proteins. However, our results suggest that due to the solvation environment within condensates, the p*K*_a_ values of the titratable residues, and therefore the pI of the sequences themselves, may shift compared to the dilute phase values. From the NCPR profiles, we expect that accounting for the p*K*_a_ shifts within the condensed phase would increase the isoelectric point of charged protein sequences relative to their dilute phase values. Therefore, if charge neutralization is the primary determinant of the interior pH of the dense phase, we would expect more basic conditions than predicted from aqueous pI values.

To validate this, we compare our expectations to existing experimental reports of the condensate pH of three protein sequences, (i) the resilin-like-polypeptide (RLP) sequence,^3^ (ii) PGL3,^12^ and (iii) FUS.^12^ Considering RLP and PGL3, we find that the measured dense-phase pH are indeed more basic than their isoelectric points with the RLP condensate having an interior pH^3^ of ∼8 (pI = 6.43) and the PGL3 condensate having a pH^12^ of ∼6 (pI = 5.1). However, in the case of FUS, the condensate pH^12^ is ∼8.5, which is more acidic than its pI of 9.4. The qualitative agreement between the RLP and PGL3 experiments and our expectations suggests that dense phase-modulated p*K*_a_ shifts may play a role in determining the interior pH of condensates, but other factors, such as ion partitioning^2,4^ and accounting for the chain conformation dependence of p*K*_a_ values,^52^ are also expected to play an important role in determining the interior pH environment of the dense phase.

### Implications and Outlook

It is useful to consider how such condensate-environment-dependent p*K*_a_ shifts and the methods used in this work may translate to biologically relevant condensate systems which are usually composed of multiple proteins and nucleic acids.^53^ Our results highlight a weak sequence-dependence of the p*K*_a_ shifts of all titratable residues in single-component systems. Whether similar behavior is observed in multicomponent condensate systems composed of protein and RNA^54–57^ or multiphasic condensates wherein each subphase has a distinct microenvironment^58^ (e.g., nucleolus^11^) is interesting to consider for future investigations.

This work demonstrates the importance of the solvation properties of the condensate microenvironment in dictating the charge states of the titratable residues. However, factors such as solution conditions,^46^ chain conformation,^52^ and condensate structure can also modulate the acid dissociation equilibria of the amino acids. To investigate the effect of protein-mediated background interactions on the p*K*_a_ shifts, we assumed similar dense phase compositions for all condensate systems. But changes to the protein sequence or solution conditions can lead to different internal environments within condensates.^46^ For instance, sequence-encoded interactions, ionic concentration, and solution pH can alter the electrostatic environment within condensates through differential ion partitioning^2,4^ and electrochemical potential changes.^3^ These factors can alter the preference for protonated vs deprotonated states. Future investigations will focus on modeling the coupling between environment-dependent p*K*_a_ shifts, internal pH conditions of the condensate, and ion-mediated effects that is required to understand the electrostatic interactions underlying the formation and stability of biomolecular condensates.^9^

Previous simulation and experimental studies in folded proteins show that the p*K*_a_ values of amino acids can be substantially altered from their model compound values based on their sequence context and conformation.^13,60^ We anticipate that in the context of IDPs and IDRs, like folded domains, sequence context, and chain conformations will lead to deviations from the p*K*_a_ shifts reported in this work. This conformational dependence will be particularly interesting in the case of phase-separating proteins where the chains are expanded in the dense phase compared to the dilute phase.^61–63^ The investigation of such effects in the context of IDPs/IDRs is particularly challenging at atomistic resolution due to their conformational plasticity. In addition to the microsecond-long time scales required to sample IDP conformational ensembles, calculating the conformation-dependent p*K*_a_ values of residues in IDRs will require the parametrization of force fields that accurately capture the conformational ensembles of IDRs^34,64,65^ within the AA CpHMD framework. Despite these challenges, AA CpHMD simulations provide a valuable tool to uncover the landscape of pH-dependent behavior of IDPs/IDRs.

## Conclusions

In this work, we use explicit solvent all-atom continuous constant pH simulations of model pentapeptides of four titratable residues (Asp, Glu, His, and Lys) to quantify the effect of the condensate microenvironment on their acid dissociation constants. We find that within the dense phase of five model condensates with different sequence features both cationic and anionic amino acids show upshifted p*K*_a_ values compared to the dilute phase.

Our results demonstrate that biomolecular condensates act as chemically distinct solvation environments that strongly stabilize protonated states of cationic residues and neutral states of anionic residues, leading to large and systematic p*K*_a_ shifts. The consistency of this effect across multiple condensate-forming sequences suggests that charge asymmetry^5,44,49^ is a general feature of condensate solvation rather than a sequence-specific anomaly. By directly quantifying how the condensate microenvironment reshapes ionization equilibria, this work reveals an unrecognized mechanism for charge regulation in phase-separated systems. These findings have broad implications for understanding how sequence composition, electrostatics, and environmental coupling jointly determine the emergent chemical properties of biomolecular condensates.^9,66^

## Supplementary Material



## Data Availability

Force field files required to run AA-CpHMD simulations, initial and equilibrated structures, and final frames from each pH replica for all amino acids in both the dilute and dense phases of the five protein condensates (SYGQ, APGVG, GRGDSPYS, GRGNSPYS, and GQGDSPYS) are publicly available on GitHub at https://github.com/shiv-rekhi/pKa_shifts_in_condensates. All simulations were performed using AMBER24 ^67^ (https://ambermd.org/AmberMD.php). The asynchronous pH replica exchange implementation^68^ and associated analysis codes^69^ are publicly available from the Jana Shen laboratory on GitLab at https://gitlab.com/shenlab-amber-cphmd

## Notes

Additional description of methods and sequences of charge-rich IDRs used for net-charge-per-residue calculations; supporting figures showing the effect of scaling protein-water Lennard-Jones interactions on the protein density profiles and dilute phase p*K*_a_ values of the titratable residues; replica walks for all residues in the SYGQ condensate; titration curves and convergence analysis for the titratable residues in the four additional condensate systems; and data investigating the impact of finite-size effects on p*K*_a_ estimates (PDF)
