# Supplementary material for: Biomolecular Condensates Act as Distinct Solvation Environments That Reshape Amino Acid pK a Values
Source: J Am Chem Soc. 2026 Jun 9;148(24):24750–8. doi: 10.1021/jacs.6c01118 (PMC13307367; doi:10.1021/jacs.6c01118)
Supplement: Supplementary file 1 [file ja6c01118_si_001.pdf]

Supporting Information for

Biomolecular Condensates Act as Distinct Solvation Environments that Reshape Amino Acid pK_a Values

Shiv Rekhi[1,*] and Jeetain Mittal[1,2,3,*]

[1]Artie McFerrin Department of Chemical Engineering, Texas A&M University, College Station, TX 77843, USA

[2]Department of Chemistry, Texas A&M University, College Station, TX 77843, USA

[3]Interdiscplinary Graduate Program in Genetics and Genomics, Texas A&M University, College Station, TX 77843, USA

[*]Correspondence – shiv197412@tamu.edu, jeetain@tamu.edu

Supporting Text

Supporting Methods

Multistep equilibration of systems for CpHMD simulations

All simulations used a cutoff of 9.0 Å for short-range nonbonded interactions, and long-range electrostatic interactions were computed using Particle Mesh Ewald[1] (PME).

The system was then minimized for 3000 steps (1000 steps steepest descent, followed by conjugate gradient) with the proteins harmonically restrained with a force constant of 50 kcal/mol/Å^2. Following this, a series of equilibration steps were carried out to gradually release the restraints on the protein, increase the temperature to the target value of 300K, and increase the time step to 4 fs. First, we simulated the system for 5000 steps with a time step of 0.5 fs at 100K and using the Langevin thermostat (gamma=1.0 ps^{-1}). Restraints were maintained on the protein with the same force constant of 50 kcal/mol/Å^2. In the second step, the time step was increased to 1 fs with the temperature maintained at 100K, and protein restraints maintained at 50 kcal/mol/Å^2. The third step involved heating the system from 100K to 300K, increasing the time step to 2 fs and reducing the force constant on the restraints to 25 kcal/mol/Å^2. In the fourth step, the system was simulated for 10000 steps at a temperature of 300K with a time step of 4 fs. Pressure was maintained at 1 bar and temperature at 300K using the Monte Carlo Barostat and the Langevin thermostat. Following the four equilibration steps, the system was further equilibrated on the GPU using the pmemd.cuda executable in AMBER24[2] for 100000 steps in the NPT ensemble with temperature 300K, pressure 1 bar, and a time step of 4 fs using the Langevin thermostat and Monte Carlo barostat. Following this, based on the system we are simulating we proceed as follows.

in the case of the condensate-only systems, this simulation was then continued for 250000000 steps for a total run time of 1 μs and coordinates were written to the trajectory file every 100 ps leading to 10000 frames from which densities were calculated using modules in MDAnalysis. The first 200 ns were discarded from the analysis as equilibration.

For CpHMD simulations in the dilute phase and dense phase, the system was run for 2.5 ns with pH-dependent sampling applied at neutral pH to allow the titration coordinates to equilibrate. The last frame from this simulation was extracted and used as the initial structure for the replica exchange CpHMD production simulations. In the dilute phase, the range of pH values for the different amino acids were, 1-7.5 in steps of 0.5 for Asp and Glu, 3-9 in steps of 0.5 for His, and 8-13 in steps of 0.5 for Lys. The total runtime for each window was 12.5 ns, with swaps attempted every 2.5 ps. The values of the titration coordinates were stored every 1.25 ps for analysis. Within the condensates, the pH ranges used for the asynchronous pH-replica exchange simulations were 2–9 for Asp and Glu, 5–13 for His, and 7–14 for Lys in all condensate systems simulated. We ran the simulations for a total runtime of 20 ns per window, attempting swaps every 4 ps and the values of the titration coordinates were stored every 2 ps. We discarded the first 5 ns of sampling and performed our analysis on the remainder of the trajectory for all amino acids in all condensate systems (**Fig. S6**). pK_a values and uncertainties are reported using the same protocol as detailed for the dilute phase with the only change being the use of 3 blocks of 5 ns each.

System size effects in the pK_a calculations in the dense phase

In AA CpHMD simulations carried out in AMBER without titratable water, there is a net charge within the simulation box due to the insertion of a counterion in the case of Asp, Glu, and His. This is due to the convention of adding counterions in the initial structure assuming charge states at neutral pH, though the simulations are always started from the protonated states. This leads to the application of a neutralizing background charge when using PME electrostatics which can artificially stabilize one charge form over another[3]. This effect of the neutralizing charge is expected to reduce with increasing box size. To test the role of this artefact in sampling, we calculated the pK_a value of Asp within an SYGQ condensate prepared in a larger simulation box of side 7.5 nm. The system was prepared and equilibrated following the same steps as for the other systems. The final frame from this simulation was extracted, the model pentapeptide was

inserted, and the simulations to calculate the pK_a were conducted following the steps and run parameters detailed above. Our test revealed that the mean pK_a value of Asp reduced by ~0.20 within the larger box compared to our 5 nm side system (**Fig. S7**). This suggests that while there is an effect of the size of simulation cell, the error introduced by the finite-size of the box is much smaller than the reported pK_a shifts.

<u>Sequences used for NCPR calculations</u>

DDX4-NTD: Asp + Glu = 36, His + Lys = 10, Arg = 24

MMGDEDWEAEINPHMSSYVPIFEKDRYSGENGDNFNRTPASSSEMDDGPSRRDHFMKSGFA
SGRNFGNRDAGECNKRDNTSTMGGFGVGKSFGNRGFSNSRFEDGDSSGFWRESSNDCEDN
PTRNRGFSKRGGYRDGNNSEASGPYRRGGRGSFRGCRGGFGLGSPNNDLDPDECMQRTG
GLFGSRRPVLSGTGNGDTSQSRSGSGSERGGYKGLNEEVITGSGKNSWKSEAEGGES

LAF1-RGG: Asp + Glu = 20, His + Lys = 1, Arg = 24

MESNQSNNGGSGNAALNRGGRYVPPHLRGGDGGAAAAASAGGDDRRGGAGGGGYRRGGG
NSGGGGGGGYDRGYNDNRDDRDNRGGSGGYGRDRNYEDRGYNGGGGGGGNRGYNNNR
GGGGGGYNRQDRGDGGSSNFSRGGYNNRDEGSDNRGSGRSYNNDRRDNGGDG

hnRNPA1-LCD: Asp + Glu = 4, His + Lys = 2, Arg = 10

GSMASASSSQRGRSGSGNFGGGRGGGFGGNDNFGRGGNFSGRGGFGGSRGGGGYGGSG
DGYNGFGNDGSNFGGGGSYNDFGNYNNQSSNFGPMKGGNFGGRSSGPYGGGGQYFAKPR
NQGGYGGSSSSSSYGSGRRF

RLP R-to-K: Asp +Glu = 20, His + Lys = 20, Arg = 0

GKGDSPYSGKGDSPYSGKGDSPYSGKGDSPYSGKGDSPYSGKGDSPYSGKGDSPYSGKGD
SPYSGKGDSPYSGKGDSPYSGKGDSPYSGKGDSPYSGKGDSPYSGKGDSPYSGKGDSPYS
GKGDSPYSGKGDSPYSGKGDSPYSGKGDSPYSGKGDSPYS

Supporting Figures

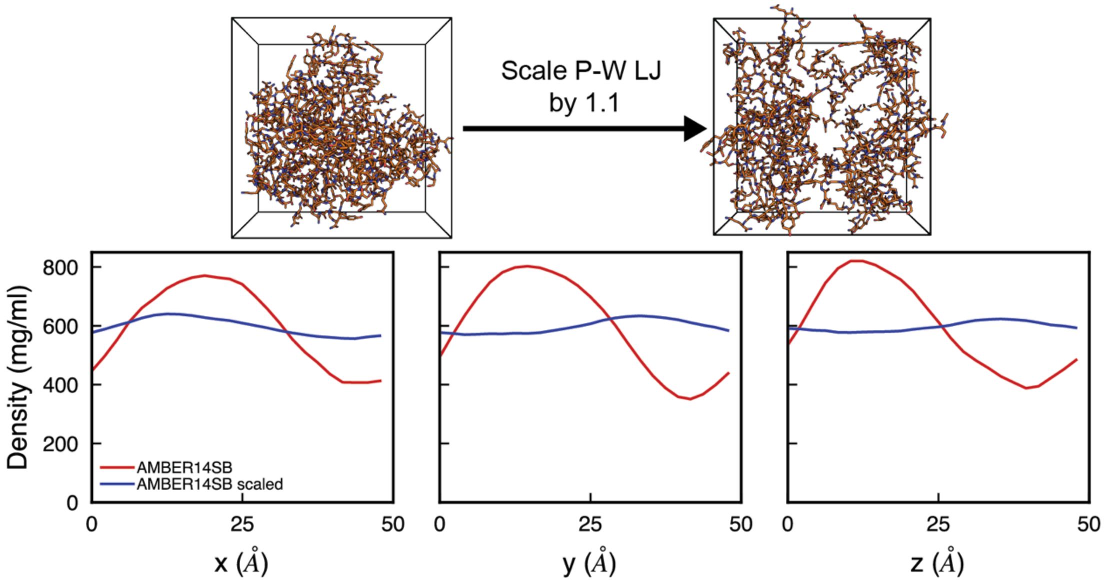

Figure S1. *Comparison of the density of protein in the x, y, and z dimensions of the simulation box for the AMBER14SB forcefield (red) and a variant of the forcefield AMBER14SB scaled (blue), where Lennard-Jones interactions between protein atoms and water oxygens are scaled by 1.1 times. Simulation snapshots above show the protein configurations at the end of a 1μs long NPT simulation. Water molecules and salt ions are hidden for clarity.*

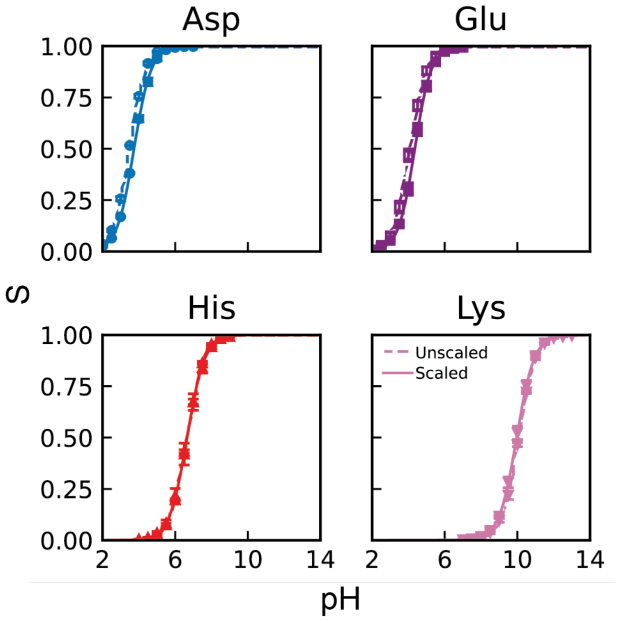

Figure S2. *Comparison of titration curves for Asp, Glu, His, and Lys in the dilute phase using the AMBER14SB protein forcefield (unscaled) and a variant of AMBER14SB where protein–water oxygen Lennard-Jones interactions are scaled by a factor of 1.1 (scaled). pK*$_a$ *values calculated from the titration curves are tabulated in Table 1.*

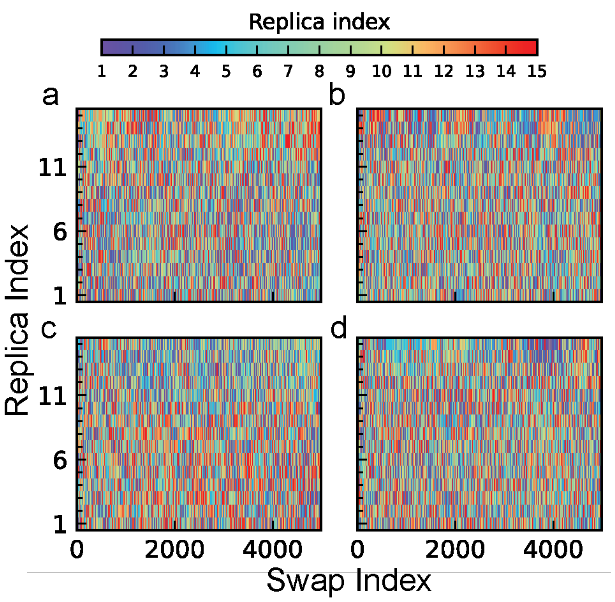

Figure S3. *Replica walks for (a) Asp, (b) Glu, (c) His, and (d) Lys in the SYGQ condensate. 500 swaps are shown, and swaps are attempted every 0.4 ns.*

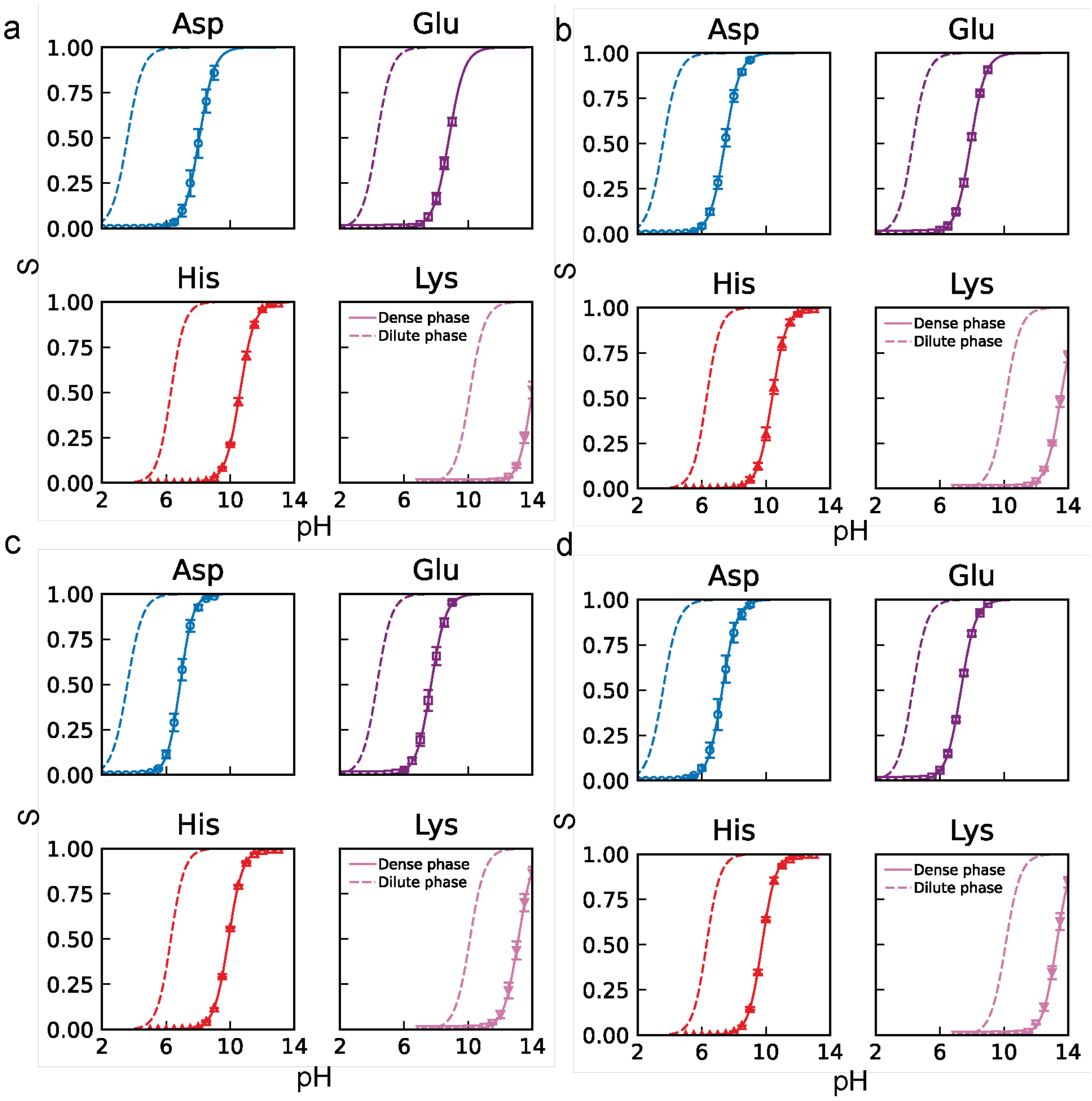

Figure S4. *Titration curves for Asp, Glu, His, and Lys in dilute phase (dashed lines) and dense phase (symbols and solid lines) of the (a) APGVG, (b) GRGDSPYS, (c) GRGNSPYS, and (d) GQGDSPYS condensates. For the dilute phase, only the HH equation fits are shown. For the dense phase, symbols represent the S values estimated from simulation and solid lines show the HH fit to the simulation data. Uncertainties on the S values from simulation are estimated as the SEM from 3 equally spaced 5 ns blocks.*

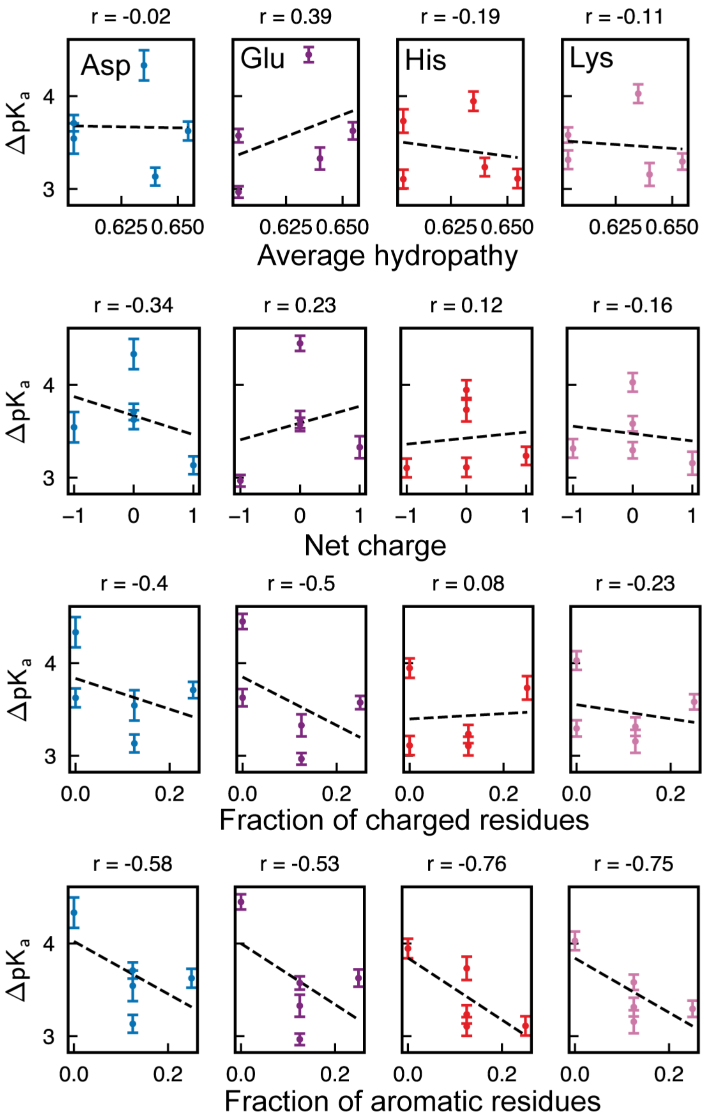

Figure S5. *pK_a shifts for Asp (column 1), Glu (column 2), His (column 3), and Lys (column 4) in the SYGQ, APGVG, GRGDSPYS, GRGNSPYS, and GQGDSPYS condensate systems plotted against four sequence-based descriptors: average hydropathy computed using the Urry hydropathy scale, the net charge of the sequence, the fraction of charged residues in the sequence, and fraction of aromatic residues in the sequence. The Pearson correlation coefficient (r) values are shown as the titles of the plots.*

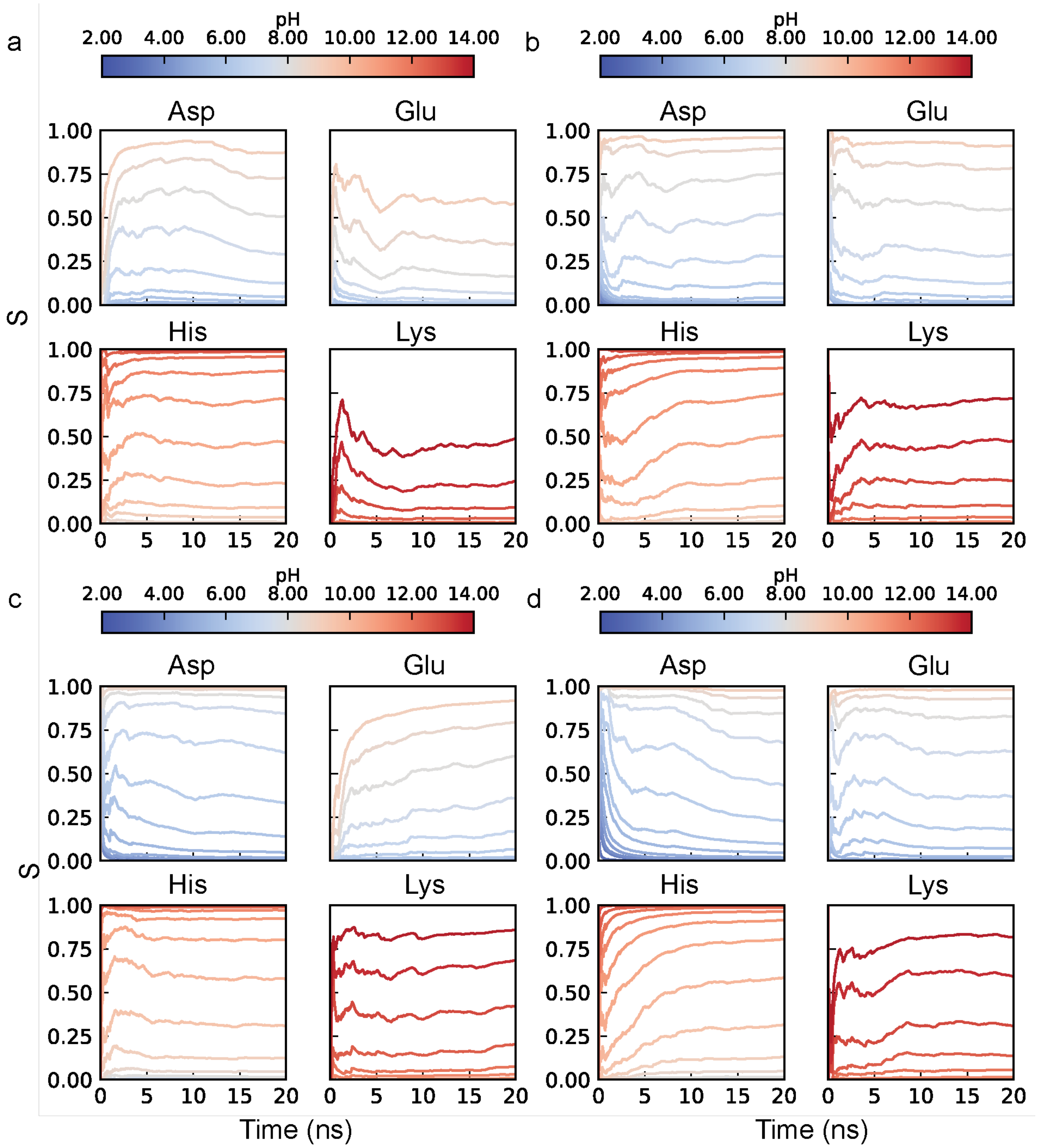

Figure S6. *Running average of deprotonated fraction (S) with time at different solution pH values for Asp, Glu, His, and Lys in the (a) APGVG, (b) GRGDSPYS, (c) GRGNSPYS, and (d) GQGDSPYS condensates.*

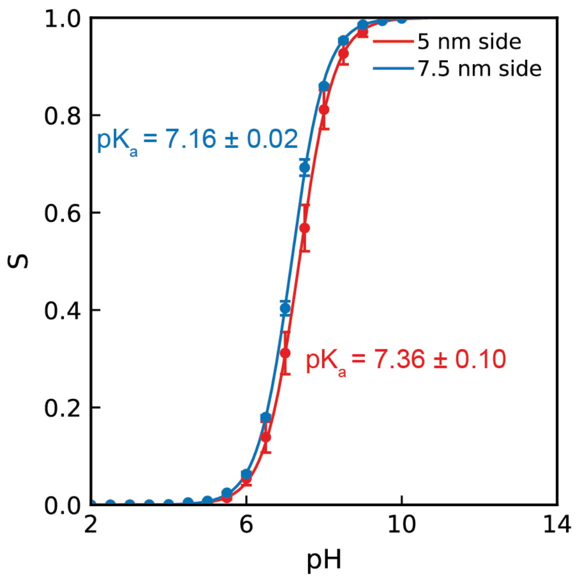

Figure S7. *Comparison of pK$_a$ values of Asp estimated within the SYGQ condensate prepared in 5 nm side cubic box and a 7.5 nm cubic side box.*

Supporting Tables

**Residue**	**pK$_a$ (Experiments)**	**pK$_a$ (AMBER14SB)**	**pK$_a$ (AMBER14SB scaled)**
Asp	3.70	3.48±0.01	3.73±0.02
Glu	4.20	4.08±0.06	4.35±0.06
His	6.50	6.64±0.01	6.65±0.10
Lys	10.40	10.06±0.01	9.96±0.06

Table S1. *Comparison of pK$_a$ values of Asp, Glu, His, and Lys embedded in the model pentapeptide (ACE-AAXAA-NHE) using the scaled and unscaled variants of the AMBER14SB forcefield. Experimental values are provided as a reference.*

References

1 Darden, T., York, D. & Pedersen, L. Particle mesh Ewald: An N log (N) method for Ewald sums in large systems. *Journal of chemical physics* **98**, 10089-10089 (1993).

2 D.A. Case, H. M. A., K. Belfon, I.Y. Ben-Shalom, S.R. Brozell, D.S. Cerutti, T.E. Cheatham, III, G.A. *et al.* Amber 2021. *University of California, San Francisco* (2021).

3 Hub, J. S., de Groot, B. L., Grubmüller, H. & Groenhof, G. Quantifying Artifacts in Ewald Simulations of Inhomogeneous Systems with a Net Charge. *Journal of Chemical Theory and Computation* **10**, 381-390 (2014). https://doi.org:10.1021/ct400626b
